# *Streptobacillus moniliformis* Endocarditis

**DOI:** 10.3201/eid1206.060069

**Published:** 2006-06

**Authors:** Nandhakumar Balakrishnan, Thangam Menon, Somasundaram Shanmugasundaram, Ramasamy Alagesan

**Affiliations:** * University of Madras, Chennai, India;; † Madras Medical College and General Hospital, Chennai, India

**Keywords:** Endocarditis, Streptobacillus moniliformis, congenital heart disease, letter

**To the Editor:** *Streptobacillus moniliformis* is a facultatively anaerobic, pleomorphic, gram-variable bacillus often seen in chains and as long unbranched filaments. It is found in the nasopharynx and oropharynx of wild and laboratory rats. Human infections result either from rodent bites (rat bite fever) or contaminated milk or other foods (Haverhill fever). The most common manifestations of infection are arthralgia, fever, and rash; endocarditis occurs as a rare complication (*1*). We report a case of *S*. *moniliformis* endocarditis in India in a patient with congenital heart disease.

An 18-year-old man was admitted to the Department of Cardiology at the Government General Hospital in Chennai, India, in November 2005, with a fever of 2 months' duration with cough, epistaxis, palpitations, and persistent joint pain. His medical history indicated congenital heart disease with a ventricular septal defect. On physical examination, his blood pressure was 100/70 mm Hg, pulse rate was 100 beats/min, and temperature was 38.5°C. Laboratory tests showed a leukocyte count of 7,600/μL, a platelet count of 127,000/μL, and an erythrocyte sedimentation rate of 70 mm/h. An electrocardiogram showed normal sinus rhythm. A transthoracic echocardiogram demonstrated a ventricular septal defect and vegetations on the septal leaflet of the tricuspid valve.

Three blood cultures were prepared, and treatment with antimicrobial drugs (intravenous penicillin G, 3 × 10^6^ U every 6 h, and gentamicin, 50 mg every 8 h for 4 weeks) was initiated. The blood cultures were incubated at 37°C in an atmosphere of 5%–10% CO_2_. Characteristic white, downy, crumblike granules were observed on the surface of the erythrocytes in all 3 cultures within 18–24 h of incubation. Characteristic puff balls were seen after 48 h of incubation. Gram-stained smears showed gram-negative bacilli in long chains. Cultures were subcultured onto 5% sheep blood agar plates and MacConkey agar plates. The plates were incubated at 37°C in an atmosphere of 5%–10% CO_2_. After 18–24 h of incubation, growth was seen on the sheep blood agar plates. Colonies were 1–2 mm in diameter, gray, smooth, and butyrous. A Gram stain of these colonies identified gram-variable, pleomorphic coccobacilli that were negative for catalase, oxidase, urease, and citrate, and did not produce indole or reduce nitrate.

Antimicrobial susceptibility testing was performed by using the Kirby-Bauer disk diffusion method according to recommendations of the National Committee for Clinical Laboratory Standards (*2*). The isolate was sensitive to penicillin G, ceftriaxone, cephalexin, amoxicillin, gentamicin, and erythromycin. The patient responded well to treatment and became afebrile within 48 h after initiation of therapy. Treatment with antimicrobial drugs was continued for 4 weeks. The blood cultures were negative when repeated after 2 weeks. The patient had an uneventful recovery and was discharged from the hospital.

Rat bite fever is a zoonosis caused by either *Streptobacillus moniliformis* or *Spirillum minus* (*1**,**3*). *S*. *moniliformis* is found in the nasopharynx of small rodents, especially rats. Rats that are carriers have no symptoms but can effectively transmit the infection by bite or through infected body fluids such as urine.

This patient had a history of living in a rat-infested area, and admitted having been bitten by a rat several months before the onset of symptoms. However, we considered it unlikely that disease contracted by a rat bite would take months to be manifested. Thus, it is more likely that he contracted the infection from food or water contaminated with rat excreta. Endocarditis is a rare complication of *S*. *moniliformis* infection, and cardiac valvular abnormalities have been reported in ≈50% of cases (*4*). This patient, however, had only a small ventricular septal defect. This is the first report of *S*. *moniliformis* endocarditis from India.
